# A fresh “slant” on modified Mitchell bladder neck reconstruction: A contemporary single-institution experience

**DOI:** 10.3389/fped.2022.933481

**Published:** 2022-09-02

**Authors:** Diana K. Bowen, Earl Y. Cheng, Josephine Hirsch, Jason Huang, Theresa Meyer, Ilina Rosoklija, David I. Chu, Elizabeth B. Yerkes

**Affiliations:** Ann & Robert H. Lurie Children's Hospital of Chicago, Chicago, IL, United States

**Keywords:** bladder neck reconstruction, neurogenic incontinence, modified Mitchell, sling, myelomeningocele, neurogenic bladder, urethral lengthening

## Abstract

**Introduction:**

Patients with neurogenic urinary incontinence due to an incompetent outlet may be offered bladder neck reconstruction, but the quest for the perfect surgical-outlet procedure continues. Our aim was to characterize continence and complications after modified Mitchell urethral lengthening/bladder neck reconstruction (MMBNR) with sling and to introduce a modification of exposure that facilitates subsequent steps of MMBNR.

**Methods:**

A single-institution, retrospective cohort study of patients who underwent primary MMBNR between May 2011 and July 2019 was performed. Data on demographics, urodynamic testing, operative details, unanticipated events, continence, bladder changes, and additional procedures were collected. A 2013 modification that permits identification of the incompetent bladder neck prior to urethral unroofing was applied to the last 17 patients. The trigone and bladder neck are exposed *via* an oblique low anterolateral incision on the bladder. Ureteral reimplantation is not routinely performed. Focal incision of the endopelvic fascia after posterior plate creation limits breadth of blunt dissection for sling placement. Descriptive statistics were utilized.

**Results:**

A total of 25 patients (13 females) had MMBNR with sling at a median age of 10 years [interquartile range (IQR) 8–11]. Bladder augmentation was performed concurrently in 14/25 (56%) patients. At a median of 5.0 (IQR 3.9–7.5) years follow-up after MMBNR, 9/11 (82%) without bladder augmentation and 13/14 (93%) with bladder augmentation had no leakage per urethra during the day without further continence procedures. Of the three patients with persistent incontinence, two achieved continence with bladder wall Botox injection (overall continence 24/25, 96%). New and recurrent vesicoureteral reflux was noted in five patients and one patient, respectively. Two patients required subsequent bladder augmentation for pressures and one other will likely require it. None have required bladder neck closure or revision.

**Conclusion:**

MMBNR with sling provides promising continence per urethra in neurogenic bladder with low need for secondary continence procedures. Ongoing modifications may achieve elusive total continence.

## Introduction

Surgical management of incontinence requires complex decision-making to balance risk and success. An ideal outlet procedure to create reliable continence in individuals with neurogenic bladder related to spinal dysraphism has unfortunately remained elusive despite numerous iterations of continence procedures. Bulking agents, slings, artificial sphincters, various bladder neck and urethral narrowing procedures, and bladder neck closure have been employed, each with a different level of invasiveness, success rate, and commitment/long-term risk for the patient.

At our institution, the preferred technique for neurogenic bladder evolved over time. Since 2011, the modified Mitchell urethral lengthening bladder neck reconstruction (MMBNR), primarily described by Mitchell for exstrophy-epispadias, has been our preferred approach for neurogenic bladder in spinal dysraphism (1). The approach involves unroofing the proximal urethra and extending full-thickness incisions into the trigone to create a longer, narrower plate for tubularization. Ureteral reimplantation is not necessarily required in neurogenic bladder as the ureters are typically orthotopic on the trigone. Based upon the more favorable continence described by Snodgrass and coauthors with the Mitchell modification with sling vs. sling alone (2), we have placed a sling in nearly all MMBNR cases to elevate and coapt the reconstructed bladder neck.

In contrast to the initial description where the unroofing begins with a transverse incision over the proximal urethra, in neurogenic bladders we have found that we might enter the urethra more distal than intended and therefore potentially too close to the ejaculatory ducts. In 2013, we developed an additional modification for the initial entry into the urethra specifically for patients with neurogenic bladder, in whom the true bladder neck may not be apparent after anterior dissection of the bladder and proximal urethra.

The aims of this study were to characterize continence and secondary procedures after MMBNR with sling and to introduce the modification that we have found to be more comfortable in the setting of bladder neck incompetence in neurogenic bladder.

## Materials and methods

Our study was carried out under IRB # 2020-3398. We performed a retrospective review of all patients who underwent primary MMBNR for urinary incontinence, as described above, at a single institution from May 2011 to July 2019. All patients had a primary diagnosis of neurogenic bladder and had persistent incontinence on their medical program. Patients were excluded if adequate operative detail or follow-up documentation was not present. Demographic information, primary and secondary diagnoses, ambulatory status, and bladder management strategy were captured. Preoperative and postoperative urodynamic tracings and images were analyzed and pertinent data points collected, including cystometric capacity (capacity/estimated bladder capacity for age) both median end-fill pressure (EFP) and groupings of EFP based on the National Spina Bifida Patient Registry risk classification system (3). Operative notes were re-reviewed by the surgeon to confirm that the procedure was MMBNR with sling. Concomitant ureteral reimplantation, enterocystoplasty, and type of sling material were detailed. Postoperative emergency department (ED) visits or hospitalizations, as well as complications specific to MMBNR, were recorded. All subsequent urologic procedures performed after the index procedure were collected, including urodynamics and imaging data.

The primary outcome of our study was urinary continence after MMBNR, stratified by concomitant augmentation. Continence was strictly defined as no daytime leakage per urethra on regular clean intermittent catheterization (CIC) every 3–4 h. Secondary outcomes were postoperative complications and reoperation for continence. Descriptive statistics were utilized with medians and interquartile ranges (IQR).

## Description of technique

The procedure involves narrowing the bladder neck and proximal urethra and lengthening its full thickness into the trigone. A sling is ultimately placed around the urethra for additional coaptation.

### Initial incision

The distalmost extent of the urethral tailoring is determined by first identifying the true bladder neck. In the neurogenic population with incompetent bladder neck, the balloon may pull into the distensible bladder neck, guiding the surgeon too distal when selecting the initial transverse incision. Narrowing and tubularization of the mid-prostatic urethra in males puts the ejaculatory ducts at risk.

**Figure 1:**
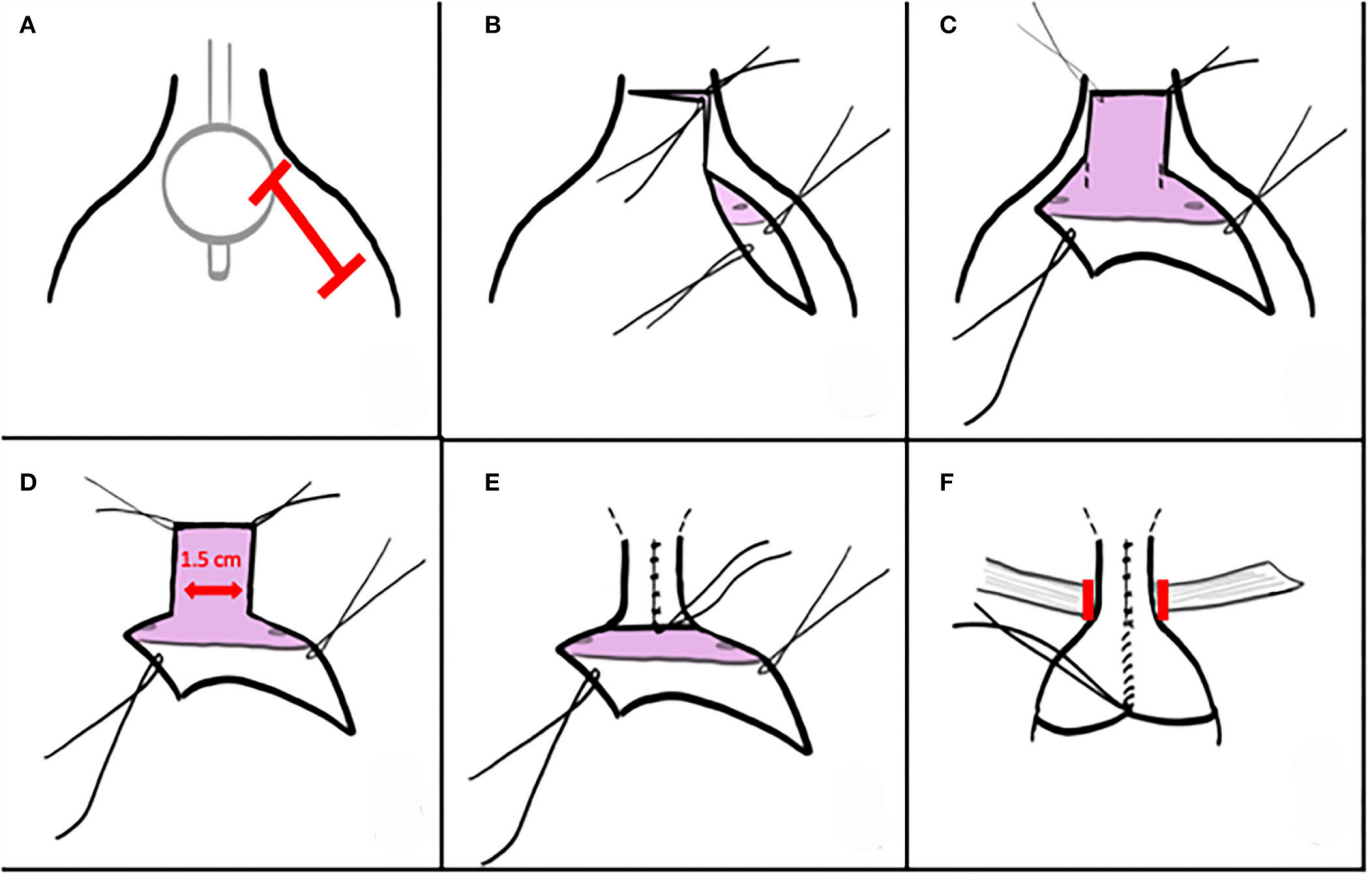
Described as part of the “Materials and methods” section.

Low oblique incision after palpating the balloon allows direct visual confirmation of the trigone and true bladder neck to guide the incision along the anterolateral proximal urethra (Figure 1A). Distance incised distally to the true bladder neck is ~1 cm. A PDS traction suture is placed distally and can be used for the distalmost urethroplasty suture. The unroofing incision is then continued transversely and then up the left anterolateral proximal urethra (Figure 1B). These incisions expose a nearly flat plate or trough of proximal urethra. A 1.5-cm-wide strip is marked on the trigone, extending a centimeter or more into the bladder but being mindful of impingement upon orthotopic ureteral orifices. With cranial traction on the urethral flap and anterior bladder wall, full-thickness incisions are created into the trigone (Figures 1C,D). Investing tissues are swept off the posterolateral bladder wall as the incision progresses, with the goal of protecting neurovascular tissue as well as Wolffian structures in males.

### Endopelvic fascial incision and dissection posterior to urethra/bladder neck

Unlike traditional longitudinal incision of the endopelvic fascia to permit blunt dissection and passage of a right angle behind bladder neck or urethra, in this case the endopelvic fascia is left attached to the urethral plate for the majority of the procedure. Either just prior to or right after the full-thickness tubularization of the urethral plate, the endopelvic fascia is focally incised. Dissection of the short distance behind the tubularized plate is performed bluntly.

### Tubularization

The posterior plate is closed full thickness over an 8 or 10 Fr Foley, beginning proximally with interrupted PDS sutures (Figure 1E). Cranial traction allows exposure of the more distal tissues and often elongates the urethroplasty as well. A second layer is not permitted nor required. A 5 or 8 Fr feeding tube is pulled in *via* the Foley or placed over a wire to serve as a splint during early healing. This tube exchange permits a smaller caliber splint, avoids irritation from a balloon on the trigone, and avoids trauma if inadvertently tugged. The tube is secured to the bladder wall or suspended in the bladder and tied to a sterile button on the abdominal wall.

### Ureteral reimplantation

As the majority of ureters are orthotopic in neurogenic bladder and are not in the section for tubularization, reimplantation is not routinely required with MMBNR. Ureteral reimplantation is required if the ureters insert ectopically near the full-thickness strip of trigone for tubularization. If MMBNR/sling is performed without concomitant bladder augmentation, existing reflux will be corrected. The decision for reimplantation in the setting of MMBNR/sling with concomitant bladder augmentation is customized based upon timing and grade of reflux and the bladder dynamics.

### Placement of sling

A sling is placed into the space dissected behind the reconstructed bladder neck and urethra after focal incision of the endopelvic fascia described above (Figure 1F). In our series, the majority were *in situ* rectus fascial slings, elevated as a strip longitudinally and left attached distally. The sling is secured into the Penrose drain and drawn beneath the bladder neck and urethra. It is then tensioned and secured to the contralateral fascial margin and then passed back across and secured at its origin with permanent suture. When utilized, free graft slings suspend the reconstructed bladder neck and urethra with sutures tied anterior to the fascia.

### Additional procedures

Importantly, a continent catheterizable channel must be created in each patient having MMBNR/sling and the patient and family understand they will no longer catheterize per urethra, possibly even in the case of emergent difficult access to the channel. The decision of whether to perform bladder augmentation is individualized based upon bladder dynamics and patient/family goals through the process of shared, complex decision-making. When bladder augmentation will not be performed, the channel is typically implanted *via* extravesical approach. If bladder augmentation will be performed, the anterior bladder wall should be closed in two running layers for ~2 cm prior to anterior anastomosis with the intestinal patch. This approach may minimize risk of leak or fistula at the bladder neck. Favorable capacity and filling pressure and commitment to continuing anticholinergic medications are required for the consideration of MMBNR/sling without augmentation.

### Postoperative drainage

The bladder is drained by suprapubic tube and catheter in the continent channel. The urethral catheter is capped unless needed for emergency drainage. Irrigations are performed twice daily and as needed. The urethral catheter is removed after 2 weeks, and the last bladder catheter removed typically at 4–5 weeks depending upon augmentation and whether ileovesicostomy is created as continent channel.

## Results

Out of 67 patients who underwent bladder neck surgery, 25 had MMBNR with sling and were eligible for inclusion. Patients with incomplete records or other types of bladder neck reconstruction, isolated sling, or bladder neck closure were excluded. A total of 25 patients (13 females) with a primary diagnosis of neurogenic bladder had MMBNR between 2011 and 2019 at a median age of 10 (IQR 8–11) years. Table 1 demonstrates the basic demographics of our cohort. The majority of sling types were *in situ* rectus fascia (*N* = 20; 80%), as well as three off-the-shelf fascia lata, and three small intestine submucosa (SIS; Cook Medical, IN, USA). Bladder augmentation was performed concurrently with MMBNR in 14/25 (56%) patients. Table 2 illustrates relevant preoperative urodynamic data points. Higher EFP with lower capacity was noted in the MMNBR group who underwent simultaneous augment. Of note, during preoperative evaluation, some patients who leak heavily clinically will not leak with the catheter traversing the sphincter. When continuous leakage occurs, a Foley with inflated balloon is used to occlude the bladder neck to simulate bladder neck competence (acutely). Of 11 MMBNR-only patients, five had additional measurements taken with the bladder neck occluded by balloon at the time of urodynamic testing. In this group, we observed a median increase in detrusor EFP of 7 cm H_2_O (range 0–25), and a wide variance in the increase in bladder capacity (median 53%, range 0–120%). In the MMBNR with augment group, bladder neck occlusion in 4 of 14 patients revealed an increase in EFP ranging from 0 to 18 cm H_2_O with cystometric capacity only marginally affected (range −3 to +35% difference).

**Table 1:** Baseline characteristics.

	***N*** **= 25**
**Sex**	
Male	12 (48%)
Female	13 (52%)
**Ambulation status**	
Ambulatory	21 (84%)
Wheelchair	4 (16%)
**Diagnoses** ^*^	
Myelomeningocele	20
Lipoma	4
Imperforate Anus	1
Caudal Regression	2
VATER	2
**Age at surgery, in years, median (IQR)**	10 (8, 11)
**Simultaneous augment**	
Yes	14 (56%)
No	11 (44%)
Length of follow-up, in years, median (IQR)	5.0 (3.9, 7.4)

* Four patients with overlapping diagnoses.

**Table 2:** Preoperative urodynamic characteristics stratified by MMBNR with simultaneous augment vs. no augment.

	**Overall**	**MMBNR without augment**	**MMBNR with simultaneous augment**
*N*	25	11	14
**NSBPR risk classifications by detrusor EFP^‡^**, ***N***			
Safe, not normal (<25 cm H20)	15	9	6
Intermediate (25–40 cm H20 or NDO)	6	2	4
Hostile (> 40 cm H20)	4	0	4
Median EFP, cm H20 (IQR)	16.5 (7.5, 30.5)	13 (2, 24)	22 (11, 42)
Median bladder capacity^*^	72.20%	93.80%	64.30%

* Measured bladder capacity/expected bladder capacity [(Age + 2) × 30].

‡ NSBPR, National Spina Bifida Patient Registry; EFP, end-fill pressure.

All medians given with interquartile range.

### Primary outcome

**Figure 2:**
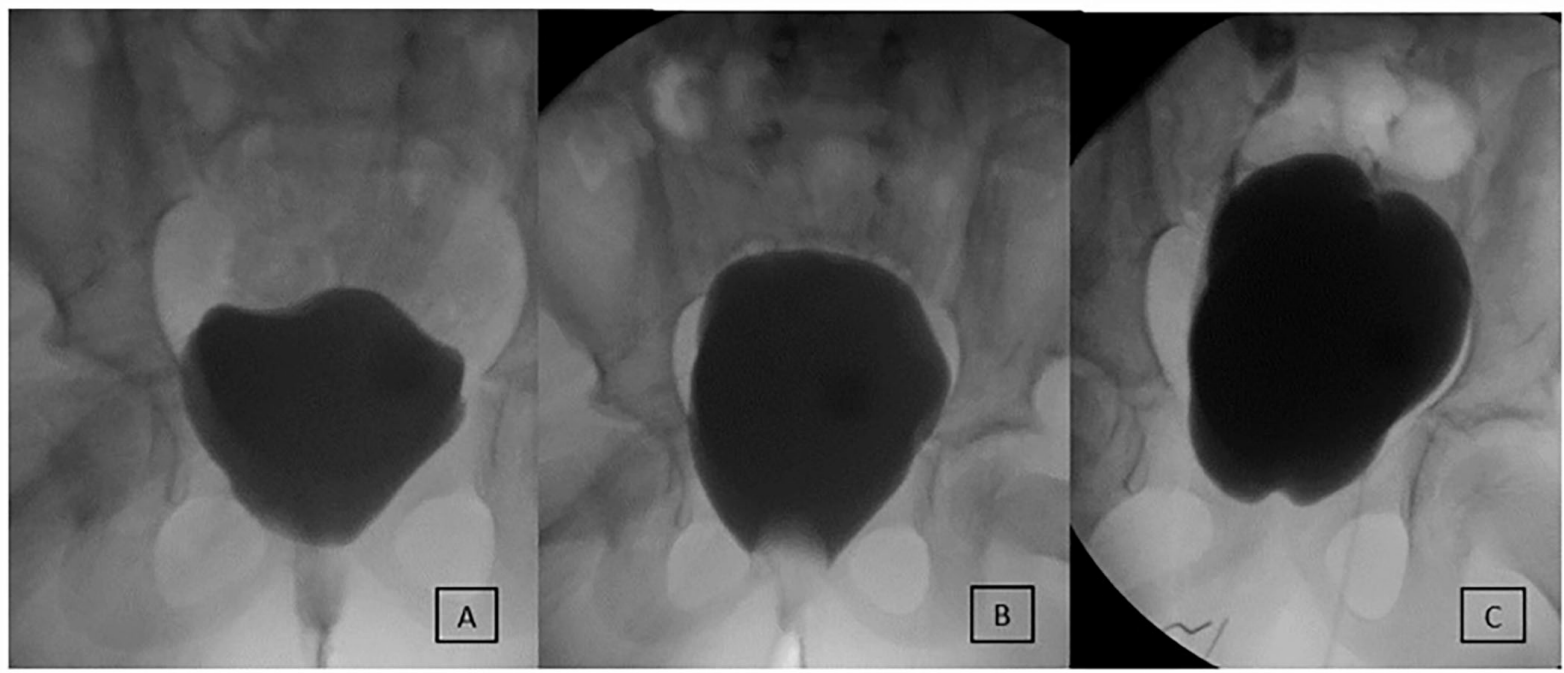
Evaluation and outcome of a 7-year-old female patient with MMBNR and sling. **(A)** Preoperative video urodynamics demonstrates early large leak at 40 ml and 2 cm H_2_O without neurogenic detrusor overactivity (NDO). **(B)** After bladder outlet occluded with a Foley balloon, the capacity reaches 270 ml with EFP 8 cm H_2_O. MMBNR, *in situ* rectus sling, and continent catheterizable channel were performed, and she was maintained on oral anticholinergics. **(C)** First postoperative urodynamics demonstrate closed and elevated bladder neck and *de novo* right grade 3 vesicoureteral reflux (VUR) at 50 ml and left grade 2 VUR at 280 ml, with end-fill pressure (EFP) 24 cm H_2_O. She remains fully continent then and at last follow-up without bladder augmentation or treatment of remaining grade 2 VUR.

**Figure 3:**
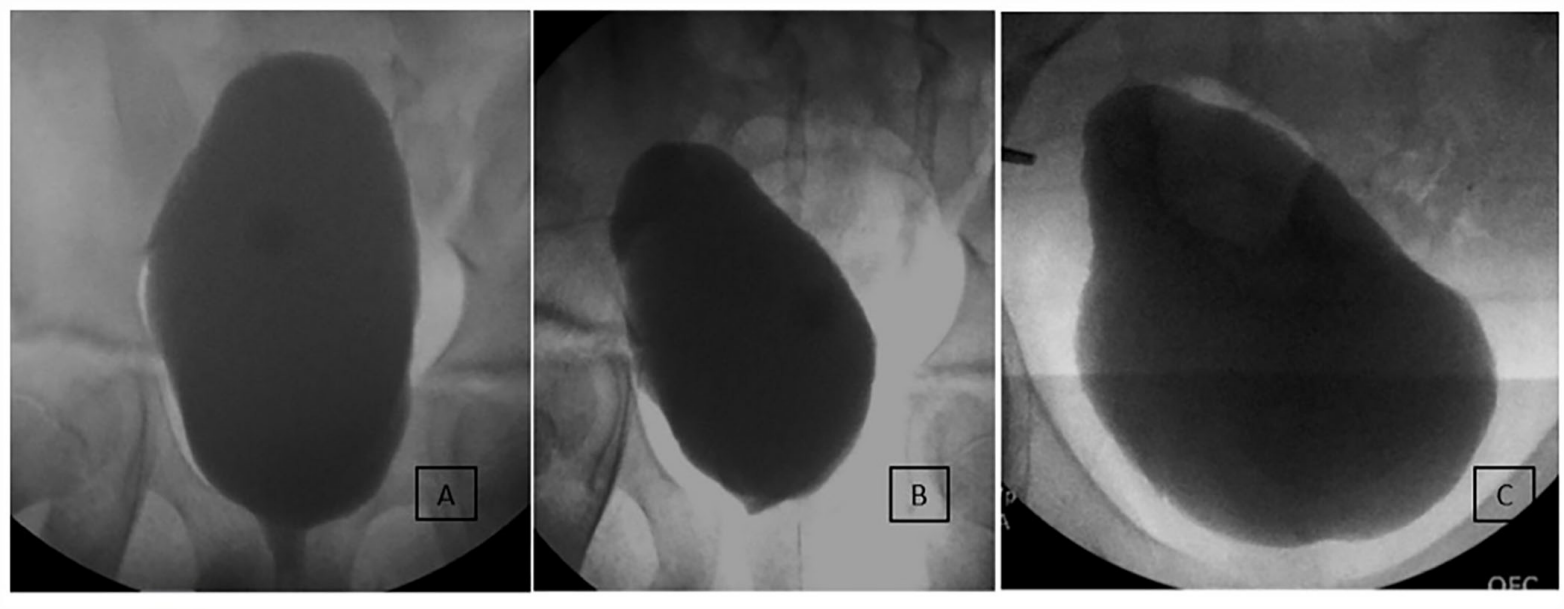
Long-term imaging of an incontinent 10-year-old male patient undergoing MMBNR with sling only. **(A)** Preoperative imaging demonstrated increasing bladder neck incompetence with filling but with sphincter effectively occluded by catheter during test. Capacity was 650 ml with 180% estimated bladder capacity (EBC), EFP 13 cm H_2_O, no NDO or VUR observed. Despite a large capacity and adherence to a CIC regimen, the patient experienced bothersome urinary incontinence and desired further treatment. **(B)** First postoperative urodynamics show mildly beaked bladder base but no leak or VUR. Capacity 350 ml (90% EBC) and EFP 10 cm H_2_O on anticholinergics. **(C)** Nine years after MMBNR with sling, he is continent per urethra without adjunctive procedures and has leakage per continent channel only when noncompliant with CIC and oral anticholinergics.

**Figure 4:**
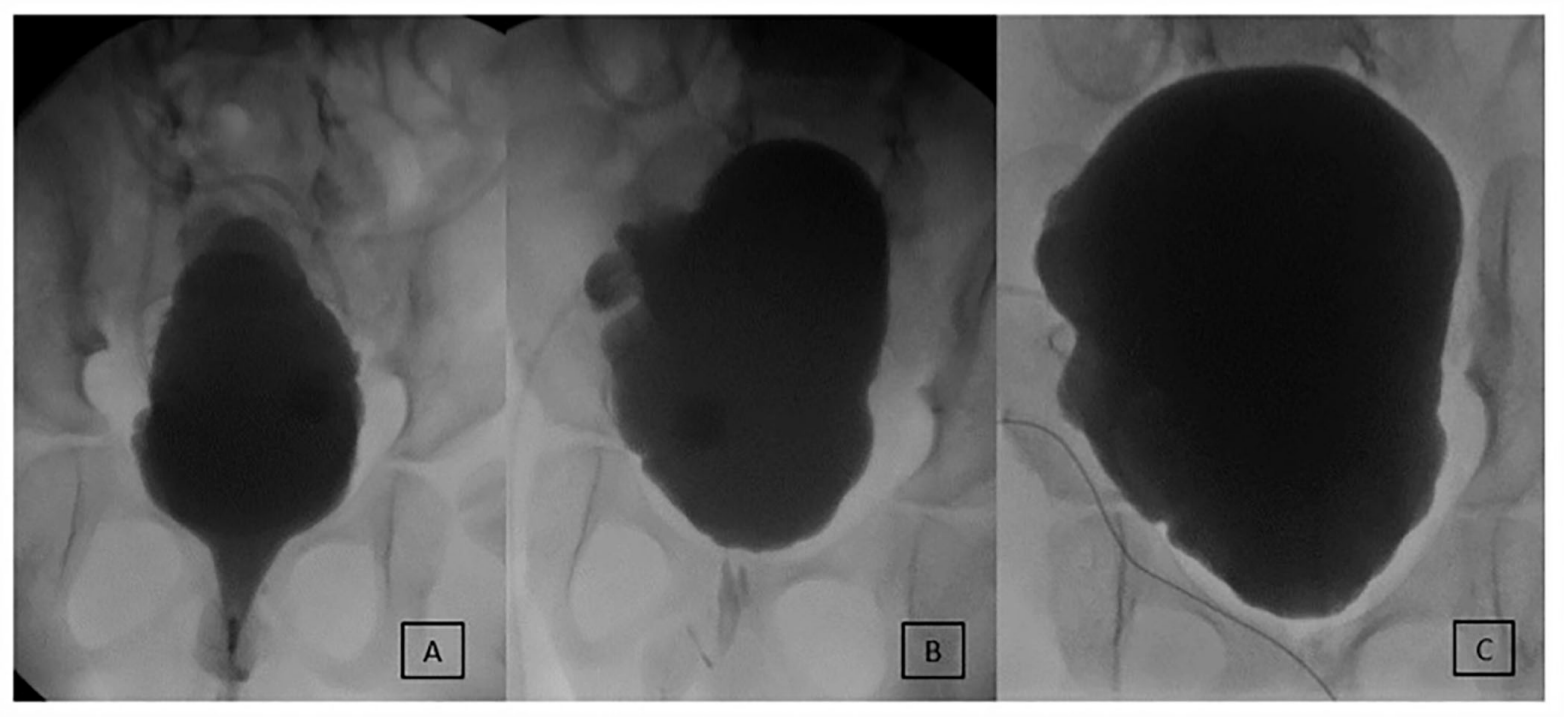
Evolution of early incontinence in an 8-year-old male after MMBNR with sling and augmentation. **(A)** Preoperative testing shows an open bladder neck and leak point pressure of 36 cm H_2_O at 220 ml. No NDO observed. **(B)** 8 months postoperatively, intermittent incontinence occurs per urethra clinically and on first urodynamics. Leakage occurred at 225 ml with NDO of 25 cm H_2_O overlying mildly impaired compliance. Botox injections to the bladder wall resolved incontinence. **(C)** Now 4 years postop, bladder neck appears competent and elevated. He is continent daytime and has scant moisture per urethra overnight with no additional procedures. He does remain on anticholinergics to manage NDO.

At a median of 5.0 (IQR 3.9–7.5) years follow-up after MMBNR, 9/11 (82%) without bladder augmentation and 14/15 (93%) with bladder augmentation had no leakage per urethra during the day without further continence procedures (Table 3). A total of 23 patients remain on anticholinergic therapy at the time of last visit, the majority on oxybutynin (96%). Of the three patients with persistent incontinence, two have achieved continence after MMBNR with bladder BTX injections (overall continence 24/25, 96%). The third patient is not bothered by their mild residual incontinence. Figures 2–4 illustrate representative preoperative and postoperative cystogram results.

**Table 3:** Continence per urethra stratified by concurrent augment.

	**Augment**	**No augment**
Patients, *n*	14	11
**Day**		
Completely dry	13	9
Leak	1^*^	2^*^

* One patient in each group dry with Botox.

### Secondary outcomes

No patients have required bladder neck closure or open revision. No injections of Deflux or other bulking agent were performed at the bladder neck after MMBNR. Seven patients have reported stomal incontinence at one point and one patient underwent Deflux to the Monti channel for leakage. For nocturnal continence, three patients in the augment group report urinary leakage at night, and none in the MMBNR with sling alone group. Of note in those that are dry, one augment and four non-augment patients use an overnight Foley.

Overall, eight of 25 patients received bladder BTX after MMBNR at median 2.3 years (IQR 1.1–4.2). Three augmented patients have undergone BTX, one for sensory urgency, one for incontinence related to bladder contractions, and one with bladder discomfort, low-amplitude contractions, and low-grade vesicoureteral reflux (VUR). The indications for Botox in non-augmented patients were either concerning bladder pressures (*n* = 5) or concerning pressures with leakage (*n* = 1) as described above. Two of these patients required subsequent bladder augmentation for pressure concerns with upper tract changes or symptoms. Another, currently preferring serial Botox, will likely progress to augmentation in the future. Of the patients who had MMBNR without augment, pre- and postoperative urodynamic findings were compared (Table 4). The median time to postoperative urodynamics was 4.5 months (IQR 4–6; range 2–10 months). Higher median EFP (13 vs. 23 cm H_2_O) was noted but no change in the number of patients with detrusor overactivity. Stratifying by National Spina Bifida Patient Registry (NSBPR) risk classifications using EFP, four patients were upstaged to a higher risk classification with one now classified as hostile. Both patients with secondary bladder augmentation were initially “safe not normal” (<25 cm H_2_O EFP). One progressed to “hostile” on first postoperative urodynamics and was managed with Botox successfully for a time. One had an episode of sepsis after brief lapse in catheterization regimen.

**Table 4:** Urodynamics pre- and postop in patients with no augment^*^.

	**Preop**	**Postop**
Median Capacity, mL	225 (120, 324)	250 (180, 380)
Median EFP, cm H20	13 (2, 24)	23 (15, 35)
Detrusor overactivity, *N*	1	1
**NSBPR risk classification** ^‡^		
Safe not normal (<25 cm H20)	9	7
Intermediate (25–40 cm H20 or NDO)	2	3
Hostile (>40 cm H20)	0	1

* *N* = 11 patients.

EFP, end fill pressure.

‡ NSBPR, National Spina Bifida Patient Registry.

Only three patients required ureteral reimplant at the time of surgery. New VUR was noted in five patients, two with concurrent augment and three without. One of these patients had recurrent reflux on one side but *de novo* reflux on the contralateral side after MMBNR. That patient went on to augmentation cystoplasty with unilateral reimplant. One teen developed the reflux 7 years after MMBNR in association with deterioration of bladder pressures and underwent bladder augmentation. No other patients have required antireflux surgery for VUR.

Complications related to the MMBNR procedure were low. Nine patients presented within 30 days with complications managed conservatively: superficial wound dehiscence (two), urinary tract infection (two), catheter drainage issues (two), and other (seizure, *Clostridium difficile* infection). One patient with concurrent augmentation, MACE, and Mitrofanoff returned to the operating room (OR) on postoperative day 6 for partial small bowel obstruction by the form of internal hernia and had externalization of his ventriculoperitoneal shunt at that time. He developed catheter malfunction with urinoma and leak from bladder neck with return to the OR for catheter manipulation and penrose drain placement. Leakage resolved and all tubes were removed by 6 weeks postop.

## Discussion

While the goal after bladder neck reconstruction in bladder exstrophy may be to void per urethra with continence, volitional voiding is not an option for neurogenic bladders that need surgical intervention. In neurogenic bladder with bladder neck deficiency, an outlet procedure would ideally provide day and night continence while maintaining upper tract integrity and reliable access for catheterization of the urethra. The “perfect” bladder neck procedure has unfortunately been difficult to achieve despite numerous modifications of various techniques. In our experience, with careful attention to bladder dynamics and use of Botox for pressure-related changes that do not mandate augmentation, our continence rate was 96% (24/25). No patients have had to undergo revision of the bladder neck or closure.

Bladder neck sling alone may be excellent for some individuals but fail to provide reliable continence in others. Gender and ambulatory status have been identified as factors contributing to success or failure, but there are no doubt various anatomical factors, bladder dynamics, patient factors, and technical aspects that result in different continence prospects (4). Techniques that narrow and/or lengthen the urethra have variable reported continence rates as well. Additional compression or elevation by a sling may enhance continence. Once the bladder neck procedure progresses beyond sling alone, most surgeons routinely create a continent catheterizable channel to ensure reliable access and minimize trauma to the continence mechanism.

Differences in reported success rates in the neurogenic bladder population are in part due to diverse patient complexity and their goals for surgical intervention. Further complicating interpretation of outcomes is both the nonstandardized definition of continence used and the myriad of named techniques utilized for BNR: Young–Dees–Leadbetter, Kropp, Salle, and the Mitchell modification of Young–Dees–Leadbetter. Reproducing the procedure or results may be challenging using the published descriptions. That is, what one believes they are doing similarly or as an innovation may not actually be so.

Much of the literature is devoted to bladder neck slings. Misseri reported overall continence of 75% with bladder neck SIS sling (27 of 36 patients) performed with augmentation and channel creation (4). Nine patients also underwent BNR with sling and continence increased to 89% (eight of nine). The authors noted a significantly less favorable result of 40% continence among ambulatory males. The authors also note that modifying their technique to lengthen and narrow the bladder neck has improved continence in their experience. Using a rectus fascial sling instead, Castellan et al. found 88% continence in 58 patients (5). The majority were female, ambulatory status was not addressed, and all patients in this series underwent simultaneous bladder augmentation.

If continence is the priority, bladder neck sling as an isolated procedure may not yield the desired result, particularly in males. Incorporation of additional procedures unfortunately increases the lifelong surgical burden for the patient but may be required if the primary goal is complete continence. A 2019 literature review by Gargollo and White regarding bladder neck procedures in children with neurogenic bladder noted that no single technique has demonstrated consistently superior outcomes (6). Overall, open BNR series have demonstrated continence rates ranging from 50 to 85%. Noordhoff et al. found a 76% continence rate in 17 patients undergoing BNR; however, this was a combination of modified Mitchell, Pippi Salle, and Dees BNR (7). The heterogeneity of Noordhoff's study and many of those in the literature limits fair assessment of a single technique.

At our institution, the treatment paradigm has shifted toward modified Mitchell urethral lengthening bladder neck repair with bladder neck sling (MMBNR with sling). The Mitchell modification of the Young–Dees–Leadbetter BNR was described by Jones et al. in 1993, primarily in patients with exstrophy-epispadias, with observation of 82% total or improved continence and nearly all voided to completion (1). Snodgrass and Barber reported improved success of 82% in 17 Leadbetter–Mitchell cases combined with a fascial sling in neurogenic bladder vs. 46% continence in 35 cases of sling alone (2). Continence was defined as being pad-free at a mean follow-up of 13 months; 60% were still dry at longest follow-up of 55 months.

Building off the other published series, Malhotra et al. assessed 12 patients who underwent Mitchell BNR with their modification of removing a diamond-shaped wedge of the anterior bladder neck and urethra following by tubularization and a fascial sling (8). This approach is different than the original Mitchell modification as it avoids urethral lengthening proximally into the trigone and ureteral reimplantation. Success rates were reported as 33% after initial procedure and 58% after subsequent bladder neck injections for those with significant leakage. Two patients underwent bladder neck closure and two others are considering it. The authors note that the published Mitchell modification involves extensive resection and tubularization with possible ureteral reimplant; in retrospect, this extended resection is likely a necessary procedural component based on their continence outcomes. They recommend returning to the tubularization procedure as described, including using a 5 Fr catheter instead of an 8 Fr.

The current series outlines the approach that we have found successful in patients with neurogenic urinary incontinence over the last decade. We developed another modification, as illustrated in the “Materials and methods” section, that we have found useful in this population. Our series demonstrates continence advantages using MMBNR with sling. There was an equal distribution of male and female patients, as well as those undergoing concurrent augmentation, and the majority of our patients were ambulatory. Three patients experienced incontinence per urethra after MMBNR/sling, and we have performed postoperative BTX in two of them with improvement in continence. In one of the patients receiving BTX, the episodic incontinence is associated with contractions and in the other the incontinence is associated with unfavorable bladder pressures. The third patient reports 95% daytime continence after MMBNR and SIS sling. He uses an overnight catheter for continence and is not currently interested in further procedures.

Our modification of MMBNR with sling, which permits earlier appreciation of the true bladder neck position to guide the distalmost extent of the urethral unroofing, has been utilized in the last 17 cases in this series. Additionally, the evolution to minimal incision of the endopelvic fascia differs from traditional exposure for sling placement. We found this to be a comfortable dissection of the reconstructed bladder neck and urethra and may preserve more support for other pelvic organs. We are not suggesting that these modifications enhance continence beyond that observed for the initial patients in our series, but we did find the adjustments to be worthy of ongoing incorporation into our approach.

Episodic leakage per continent channel has been endorsed at some point in the clinical course in seven patients. Noncompliance with the medical program was implicated in five and the other two patients were those successful with BTX for urethral incontinence. One patient elected Deflux to the channel for false passage. No patients have had surgical revision of the stoma for incontinence.

Secondary VUR occurred in a small number with and without concomitant bladder augmentation. Three cases were low grade and asymptomatic. One patient had recurrence of previously resolved reflux and occurred bilaterally, and the other developed during the course of management of a change in bladder compliance. The latter two patients required secondary bladder augmentation. This presentation of reflux again demonstrates the heterogeneity of the population and their bladders. Nevertheless, the occurrence of secondary reflux emphasizes the need to be mindful of the proximal extent of the urethral lengthening incisions into the trigone due to possible distortion or infringement upon the ureteral orifice. It also raises the question as to whether the extent of the trigone dissection, beyond that related to increased outlet resistance, has an impact on bladder function.

The decision of whether to perform simultaneous augmentation cystoplasty is often not straightforward, and no rigid selection criteria were in place for this retrospective cohort. Preoperative urodynamic detrusor pressures and outlet appearance on cystogram are important to inform this decision, as is a holistic view of the patient medically and socially, incorporating their goals. The ability of the family to take on the risks and long-term care is also considered. We counsel patients on the known risk of subsequent augmentation after MMBNR with sling and the possibility of upper tract damage if bladder changes occur. Over half of our patients had a simultaneous augmentation (56%). In the 11 patients who did not have an augment at the time of surgery, nine were continent per urethra after MMBNR with sling. We did not see a significant difference in continence between augmented and non-augmented patients, perhaps due to very careful selection criteria and close follow-up with testing.

When there was a change in bladder dynamics, it did not often result in new incontinence, rather concerning upper tract changes in the form of VUR or urodynamic pressures were observed. Therefore, in this series, the cohort of greatest concern is not the incontinent group, but rather those who received the desired continence but still require not only consistent CIC but also anticholinergic medication, daily bladder maintenance, and long-term upper tract surveillance. Incontinence is no longer a motivator for compliance with the medical program. The responsibility is on the surgeon, patient, and family to ensure that there is a clear understanding of the long-term care needs and risks as the cost of surgical continence and that the program is followed. This may be difficult to achieve as adolescents and teens are given more responsibility for self-management and may falter. Adults may likewise be well-intentioned but under-resourced for self-management.

## Limitations

Our series contains the inherent limitations of a retrospective review with relatively small numbers. A review of records that spanned the transition from paper to electronic records also makes it possible that some data would be missing and require exclusion. As with other reconstruction series, there is heterogeneity of the population and their response to surgical interventions, compounded by periods of medical noncompliance that may or may not be earnestly disclosed. Cystoscopy per urethra has not been performed to determine whether MMBNR with sling is simply well-coapted or actually unintentionally closed. This possibility emphasizes the importance of creating and maintaining a reliable continent catheterizable channel to accompany surgical continence.

## Conclusion

MMBNR with sling provides promising continence in neurogenic bladder with low need for secondary continence procedures or secondary augmentation. Ongoing institutional re-evaluation of technique and honest reporting of results are vital to producing a reliable surgical technique for bladder outlet procedures. Most important, however, is the preparation of patient and family regarding what they can expect for outcomes and what they will be obligated to maintain during long-term surveillance of bladder and kidneys.

## Data availability statement

The raw data supporting the conclusions of this article will be made available by the authors, without undue reservation.

## Author contributions

DB, EY, EC, DC, and IR contributed to the initial idea, design, data review, data analysis, and writing and review of the manuscript. TM, JHi, and JHu contributed to the design, data extraction, and review of the manuscript. All authors contributed to the article and approved the submitted version.

## Conflict of interest

The authors declare that the research was conducted in the absence of any commercial or financial relationships that could be construed as a potential conflict of interest.

## Publisher's note

All claims expressed in this article are solely those of the authors and do not necessarily represent those of their affiliated organizations, or those of the publisher, the editors and the reviewers. Any product that may be evaluated in this article, or claim that may be made by its manufacturer, is not guaranteed or endorsed by the publisher.
